# Donor-derived cell-free DNA is associated with acute rejection and decreased oxygenation in primary graft dysfunction after living donor-lobar lung transplantation

**DOI:** 10.1038/s41598-018-33848-3

**Published:** 2018-10-18

**Authors:** Shin Tanaka, Seiichiro Sugimoto, Takeshi Kurosaki, Kentaroh Miyoshi, Shinji Otani, Ken Suzawa, Shinsuke Hashida, Masaomi Yamane, Takahiro Oto, Shinichi Toyooka

**Affiliations:** 1 Department of General Thoracic Surgery, Okayama University Hospital, 2-5-1 Shikata-cho, Kita-ku, Okayama, 700-8558 Japan; 2 Organ Transplant Center, Okayama University Hospital, 2-5-1 Shikata-cho, Kita-ku, Okayama, 700-8558 Japan

## Abstract

Donor-derived cell-free DNA (dd-cf-DNA) has been shown to be an informative biomarker of rejection after lung transplantation (LT) from deceased donors. However, in living-donor lobar LT, because small grafts from blood relatives are implanted with short ischemic times, the detection of dd-cf-DNA might be challenging. Our study was aimed at examining the role of dd-cf-DNA measurement in the diagnosis of primary graft dysfunction and acute rejection early after living-donor lobar LT. Immediately after LT, marked increase of the plasma dd-cf-DNA levels was noted, with the levels subsequently reaching a plateau with the resolution of primary graft dysfunction. Increased plasma levels of dd-cf-DNA were significantly correlated with decreased oxygenation immediately (p = 0.022) and at 72 hours (p = 0.046) after LT. Significantly higher plasma dd-cf-DNA levels were observed in patients with acute rejection (median, 12.0%) than in those with infection (median, 4.2%) (p = 0.028) or in a stable condition (median, 1.1%) (p = 0.001). Thus, measurement of the plasma levels of dd-cf-DNA might be useful to monitor the severity of primary graft dysfunction, and plasma dd-cf-DNA could be a potential biomarker for the diagnosis of acute rejection after LT.

## Introduction

Long-term survival after lung transplantation (LT) has been worse than that after other solid organ transplantations (median survival: lung 6.0 years, heart 10.7 years, liver 8.5 years)^1–3^. The major obstacle against long-term survival after LT is the occurrence of chronic lung allograft dysfunction, and for the development of chronic lung allograft dysfunction, primary graft dysfunction and acute rejection (AR) have been identified as strong independent risk factors for the development of chronic lung allograft dysfunction^4,5^. Moreover, primary graft dysfunction could cause AR through the linkage between innate and adaptive immune responses after LT^6^. For the diagnosis of AR, transbronchial biopsy is generally performed after cadaveric LT. However, after living-donor lobar LT (LDLLT), because the right and left lower lobes from two healthy donors are implanted in the recipient in the place of entire lungs, AR is commonly diagnosed on the basis of clinical and radiographic findings, as to avoid unexpected bleeding from the small lobar grafts as a result of the invasive bronchoscopic procedure^7–9^. Therefore, after LDLLT, in particular, a noninvasive diagnostic test for AR is required as an alternative to transbronchial biopsy to avoid bleeding and pneumothorax^4^, although LDLLT could provide similar survival to cadaveric LT even in critically ill patients owing to this strategy^8^.

Recently, measurement of the plasma level of donor-derived cell-free DNA (dd-cf-DNA) was shown to be useful as a noninvasive diagnostic test for AR after cadaveric LT^10^. Cell-free DNA (cf-DNA) consists mainly of 166 base-pair double-stranded DNA fragments resulting from apoptosis, necrosis or release of nuclear DNA into the circulation^8,9^. In the circulation, these fragments have a short half-life of 1.5 hours, because of rapid hepatic and renal clearance^11^. Thus, dd-cf-DNA could be a real-time monitoring biomarker of graft tissue damage after solid organ transplantation^12^, such as primary graft dysfunction and AR after LT. In addition, the usefulness of genotyping of circulating DNA released from tumors, known as “liquid biopsy,” is being investigated in the field of cancer research^13^.

Different from cadaveric LT, LDLLT is characterized by transplantation, with short ischemic times, of small lobar grafts from blood relatives or spouses. The lower degree of lung injury in the small lobar grafts might lead to some difficulty in the quantification of dd-cf-DNA after LDLLT. Moreover, the similarity of single nucleotide polymorphism (SNP) between the living donors and the recipient could complicate the identification of dd-cf-DNA in cases of LDLLT. Thus, the detection of dd-cf-DNA after LDLLT may pose a challenge, and the role of measurement of dd-cf-DNA after LDLLT remains unclear. The aim of this study was to assess the role of dd-cf-DNA measurement in the diagnosis of primary graft dysfunction and AR early after LDLLT.

## Results

### Patient characteristics

**Figure 1:**
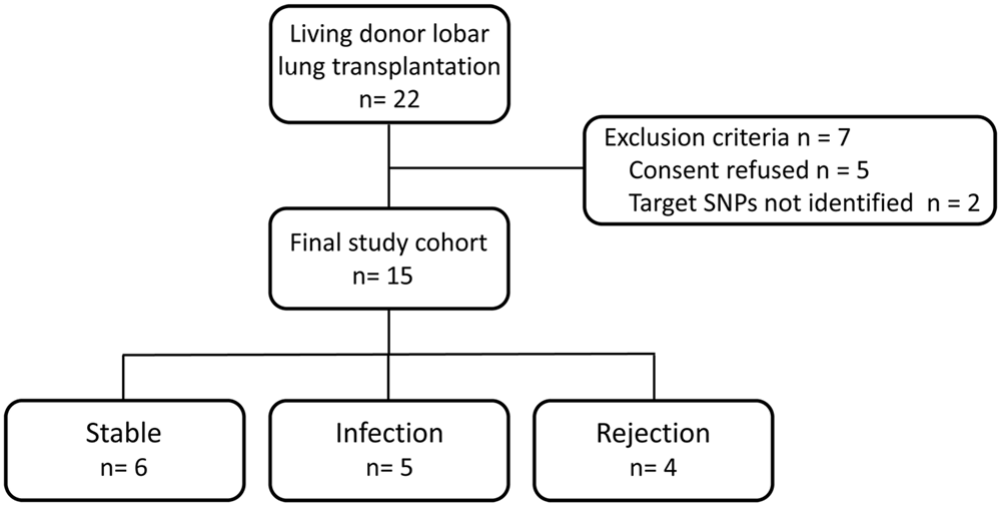
Of 22 patients who underwent living-donor lobar lung transplantation, 5 patients who did not provide consent for genotyping and 2 patients in whom SNPs could not be adequately identified between recipients and blood-relative donors, were excluded from this study. The remaining 15 patients were divided into 3 groups according to the occurrence of clinical events: the stable group (n = 6), the infection group (n = 5) and the rejection group (n = 4).

The patient characteristics of each group (Fig. 1) are shown in Table 1. The preoperative and operative characteristics were similar among the stable, infection and rejection groups. The lung graft volumes from the living donor, which was measured by pulmonary function testing and three-dimensional computed tomography, did not differ among the three groups. In the stable group, none of the patients developed any complications between postoperative day (POD) 0 and POD14 after LDLLT. In the infection and rejection groups, none of the patients developed any complications until POD 6 after LDLLT, but subsequently, infection or AR developed between POD7 and POD14. As shown in Table 2, postoperative complications developed exclusively in the unilateral transplanted lung after LDLLT. The details of targeted SNPs of each patient are shown in Table 3.

**Table 1:** Patient characteristics.

Variables	Stable group (n = 6)	Infection group (n = 5)	Rejection group (n = 4)	P-value
Age (years), median (range)	8 (2–29)	22 (6–55)	15 (10–24)	0.43
Number of the patients < 18 years (%)	5 (33.3)	2 (13.3)	2 (13.3)	0.31
Gender				0.51
Male (%)	4 (26.7)	2 (13.3)	3 (20.0)	
Female (%)	2 (13.3)	3 (20.0)	1 (6.7)	
Diagnosis				0.34
Pulmonary graft versus host disease (%)	3 (20.0)	1 (6.7)	3 (20.0)	
Interstitial lung disease (%)	2 (13.3)	3 (20.0)	0	
Pulmonary hypertension (%)	1 (6.7)	0	1 (6.7)	
Bronchiectasis (%)	0	1 (6.7)	0	
Predicted lung volume of the graft (ml), median (range)	737 (608–1041)	650 (310–1029)	860 (691–1106)	0.44
3D-CT volumetry of the graft (ml), median (range)	992 (590–1380)	776 (465–1185)	1074 (652–1259)	0.46
Lung transplant procedure				0.94
Single (%)	3 (20.0)	2 (13.3)	2 (13.3)	
Bilateral (%)	3 (20.0)	3 (20.0)	2 (13.3)	
Ischemic time (min), median (range)	124 (98–180)	133 (106–198)	78.5 (71–144)	0.21
Operative time (min), median (range)	456 (239–608)	470 (221–785)	380 (300–546)	0.52
Blood loss (ml), median (range)	395 (250–1650)	460 (180–1090)	392 (270–1210)	0.96
Intubation time (day), median (range)	2 (1–12)	5 (1–17)	2 (2–46)	0.47
ICU stay (day), median (range)	15 (8–28)	17 (5–28)	17 (14–101)	0.73

Data are presented as n, median (range) or n (%). 3D-CT; three-dimensional computed tomography, ICU; intensive care unit.

**Table 2:** Details of postoperative complications.

Patient	Group	Postoperative complication	Lung transplant procedure	Relationship with the recipient		Laterality of complication	Clinical onset of complication	Days exceeding the threshold levels of dd-cf-DNA
				Right donor	Left donor			
1	Stable	None	Single	Mother	—	None	—	—
2	Stable	None	Single	Mother	—	None	—	—
3	Stable	None	Single	Father	—	None	—	—
4	Stable	None	Bilateral	Mother	Father	None	—	—
5	Stable	None	Bilateral	Father	Mother	None	—	—
6	Stable	None	Bilateral	Father	Mother	None	—	—
7	Stable	None	Bilateral	Father	Mother	None	—	not identified
8	Infection	Pneumonia	Single	Mother	—	Right	POD 7	—
9	Infection	Pneumonia	Single	Mother	—	Right	POD 6	—
10	Infection	Pneumonia	Bilateral	Sister	Father	Left	POD 10	—
11	Infection	Pneumonia	Bilateral	Son	Wife	Left	POD 11	—
12	Infection	Pneumonia	Bilateral	Sister	Father	Left	POD 10	—
13	Rejection	Acute rejection	Single	Father	—	Right	POD 10	POD 10
14	Rejection	Acute rejection	Single	Mother	—	Right	POD 12	POD 9
15	Rejection	AMR	Bilateral	Father	Uncle	Left	POD 8	POD 8
16	Rejection	Acute rejection	Bilateral	Sister	Sister	Right	POD 8	POD 7
17	Rejection	Acute rejection	Bilateral	Father	Mother	Right	POD 6	not identified

AMR; antibody mediated rejection, dd-cf-DNA; donor-derived cell-free DNA, POD; postoperative day.

**Table 3:** Details of targeted single nucleotide polymorphism (SNP).

Patient	Group	db SNP ID	Assays ID	Chromosome location	Nucleotide variation	Minor allele frequency
1	Stable	rs1995691	C__11288969_1_	12	A/G	0.46
2	Stable	rs2567609	C___3206281_1_	20	C/T	0.432
3	Stable	rs17309	C____336443_1_	22	A/G	0.49
4	Stable	rs1995691	C__11288969_1_	12	A/G	0.46
5	Stable	rs17309	C____336443_1_	22	A/G	0.49
6	Stable	rs10277115	C__11236947_10	7	T/A	0.45
7	Stable	not identified	—	—	—	—
8	Infection	rs2567609	C___3206281_1_	20	T/C	0.432
9	Infection	rs767007	C___1023295_20	5	G/C	0.49
10	Infection	rs3790955	C__11677418_10	1	T/C	0.41
11	Infection	rs1800470	C__22272997_10	19	A/G	0.41
12	Infection	rs37972	C____970132_10	7	T/C	0.43
13	Rejection	rs1995691	C__11288969_1_	12	A/G	0.46
14	Rejection	rs3790955	C__11677418_10	1	T/C	0.41
15	Rejection	rs2301964	C___2179875_1_	8	G/C	0.47
16	Rejection	rs2567609	C___3206281_1_	20	C/T	0.432
17	Rejection	not identified	—	—	—	—

### Postoperative changes of the plasma dd-cf-DNA levels in the stable group

**Figure 2:**
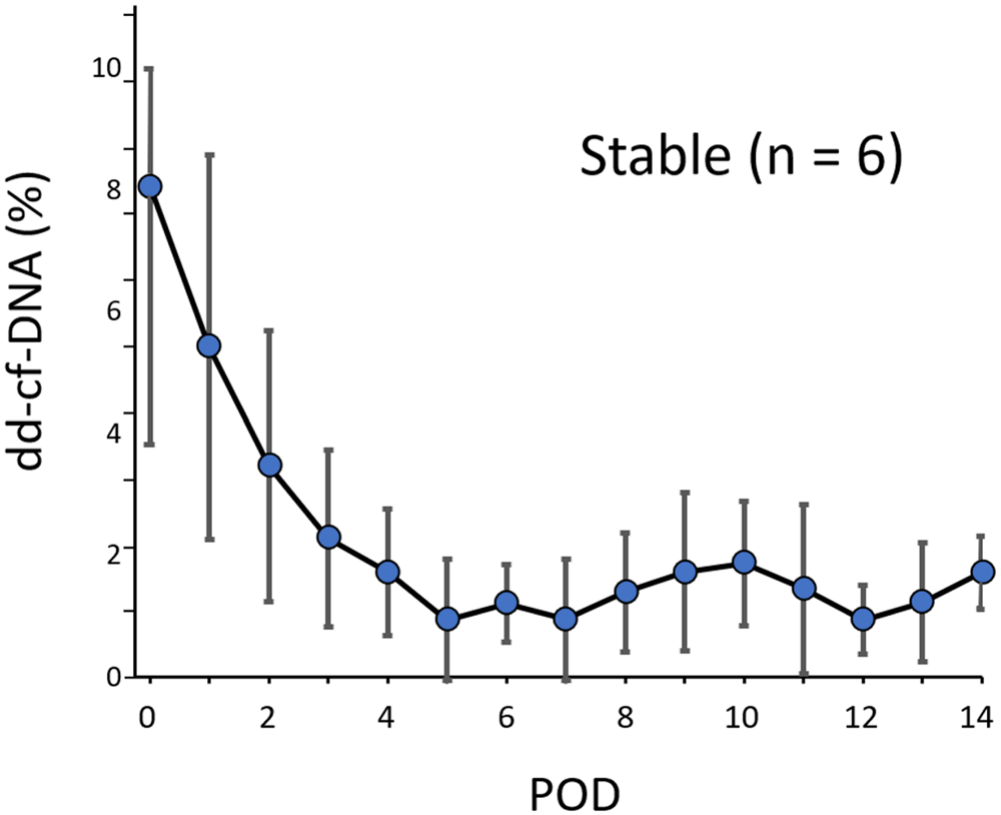
Time-course of the plasma levels of donor-derived cell-free DNA (dd-cf-DNA) in the stable group between postoperative day (POD) 0 and POD 14 after living-donor lobar lung transplantation. The circle plots represent the mean values, and the vertical bars indicate the standard deviation from the mean. The dd-cf-DNA levels were markedly elevated immediately after the transplantation, presumably reflecting ischemia/reperfusion injury, and the mean dd-cf-DNA levels decreased to 0.93 ± 0.99% in the stable group within the first 5 days after transplantation.

In the stable group, marked elevation of the plasma dd-cf-DNA levels was observed immediately after the LDLLT, with the levels subsequently decreasing, in a time-dependent manner, to a mean level of 0.93 ± 0.99% on POD 5 (Fig. 2). The dd-cf-DNA values in the stable group (n = 6) remained low thereafter, without any surges, from POD 5 to 14.

### Plasma dd-cf-DNA levels during primary graft dysfunction after LDLLT

The plasma dd-cf-DNA values on POD 0 and POD 3 after LDLLT in all 15 patients were significantly negatively correlated with the ratio of the arterial oxygen tension to the inspired oxygen fraction, or the PaO_2_/FiO_2_ ratio (P/F ratio) (POD 0: R^2^ = 0.39, p = 0.022; POD 3: R^2^ = 0.34, p = 0.046). In addition, the values between POD 0 and POD 3 were not correlated with the lung graft volume as measured by pulmonary function testing or three-dimensional computed tomography. The P/F ratio and primary graft dysfunction grade between POD 0 and POD 3 after LDLLT did not differ between the three groups.

### Plasma dd-cf-DNA levels during AR and infection after LDLLT

**Figure 3:**
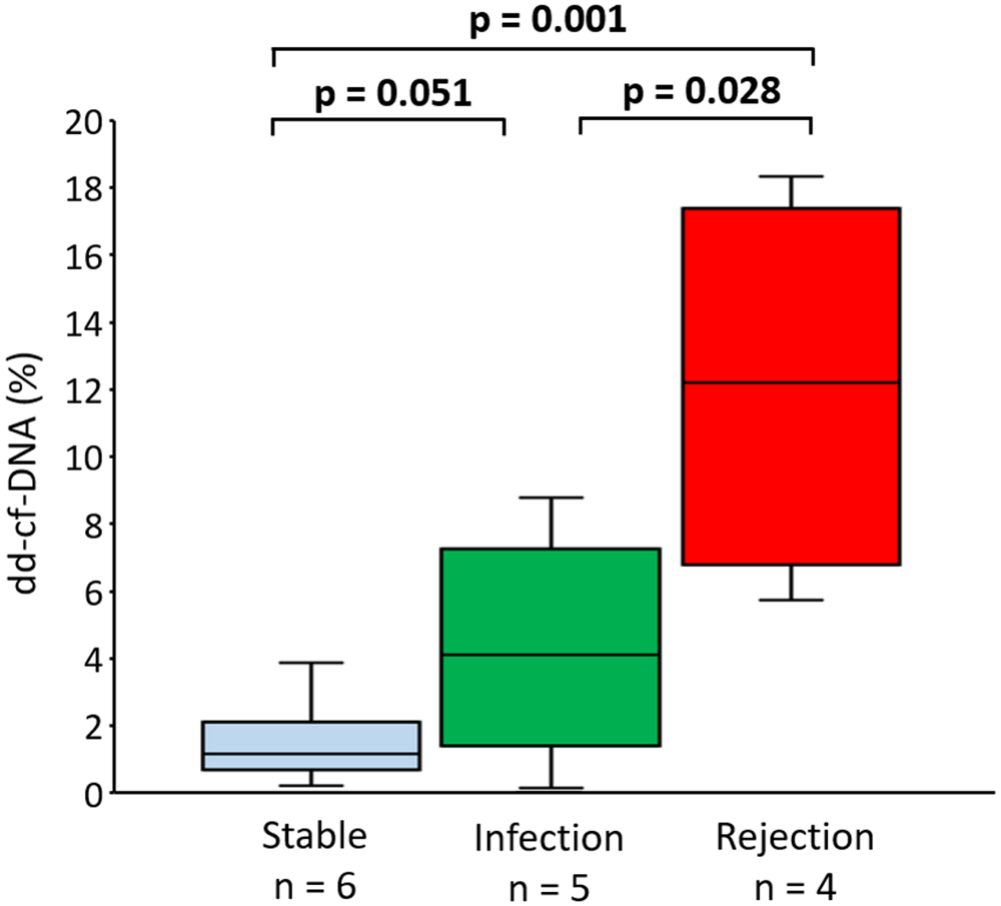
Plasma donor-derived cell-free DNA (dd-cf-DNA) levels in the stable group (Stable, n = 6) and the infection group at disease onset (Infection, n = 5), and in the rejection group at disease onset (Rejection, n = 4) between 5 and 14 days after living-donor lobar lung transplantation. The dd-cf-DNA levels are presented as median values (black line), interquartile range (box), and 5th and 95th percentiles (whiskers). The dd-cf-DNA levels in the rejection group were significantly higher than those in the infection group (p = 0.028) and the stable group (p = 0.001).

Between POD 5 and POD 14 after LDLLT, the plasma dd-cf-DNA values were maintained at a low level, with a median of 1.1%, in the stable group (Fig. 3). By contrast, as compared to the stable group, the median dd-cf-DNA levels were significantly higher in the AR group on the day of clinical onset of AR (median, 12.0%; range, 5.7%-18.5%; p = 0.001) but were not significantly different in the infection group on the day of clinical onset of infection (median, 4.2%; range, 0.1%-8.8%; p = 0.051).

**Figure 4:**
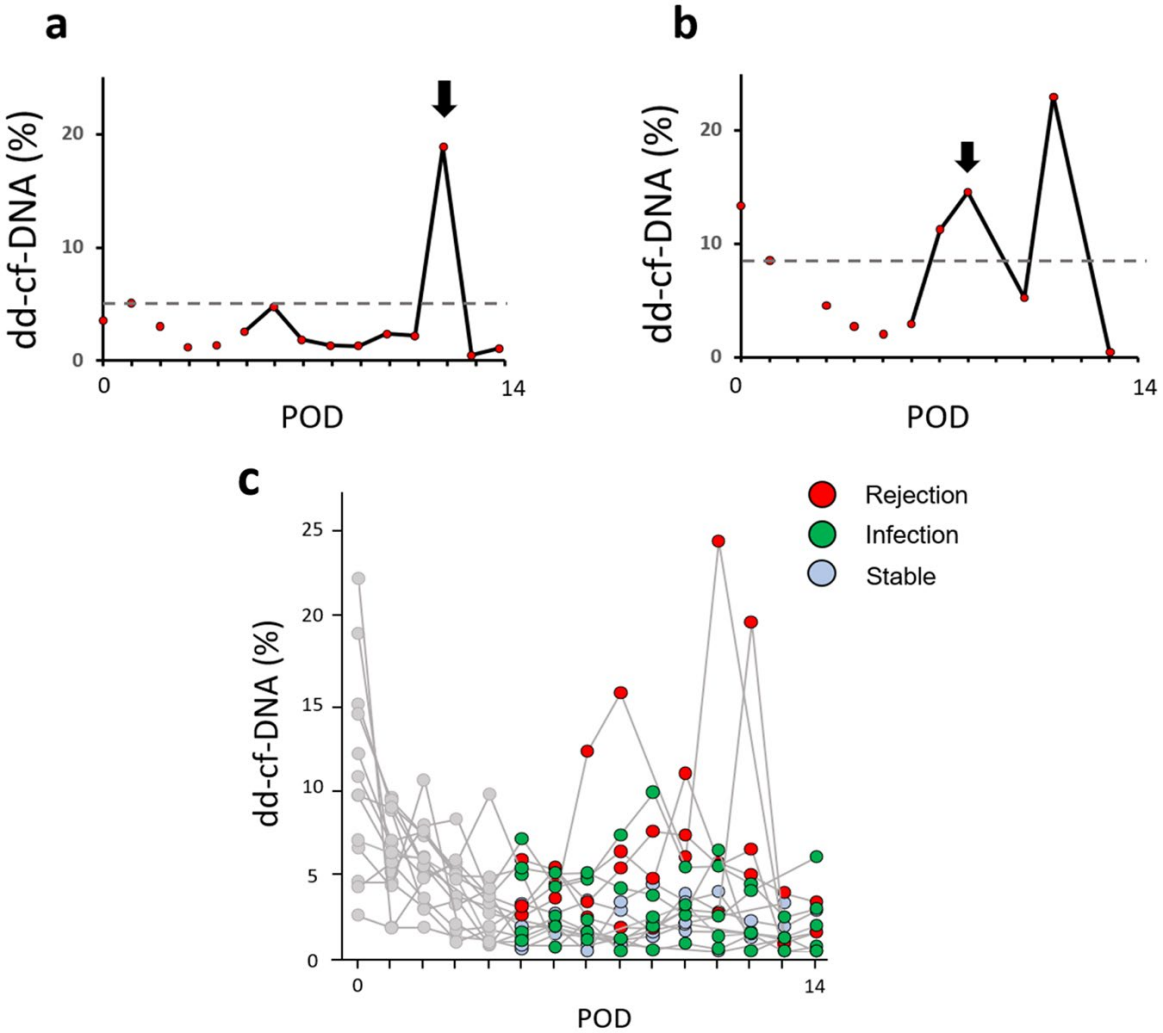
Time-course of the donor-derived cell-free DNA (dd-cf-DNA) levels in the rejection group. (**a**) A recipient who developed an episode of acute rejection on POD 12. Black arrow represents the clinical onset of rejection, and dot-line represents the threshold levels of dd-cf-DNA for the diagnosis of acute rejection. (**b**) A recipient who developed episode of antibody-mediated rejection and subsequently required lung retransplantation on POD 40. (**c**) Overview of all data collected from the rejection group (red circles), infection group (green circles) and stable group (blue circles). Data collected between POD 0 and POD 4 are shown in gray, and were excluded from the signal analysis.

The representative time-courses of the plasma dd-cf-DNA levels showed marked elevation of the plasma dd-cf-DNA levels (18.5%) from the baseline in the rejection group (Patient 14) (Fig. 4a). Moreover, in the patient with antibody-mediated rejection (AMR) (Patient 15), who required left single re-LDLLT from the mother on POD 40, the levels continued to be markedly elevated from the baseline (23.0%) despite steroid pulse therapy (Fig. 4b). For the diagnosis of acute rejection, the maximum value of dd-cf-DNA levels between POD 1 and 4 was determined as the threshold in each case in this study (Fig. 4a,b). The dd-cf-DNA levels in the rejection group exceeded the threshold prior to or at the timing of clinical onset of acute rejection as shown in Table 2, while in the infection group and the stable group, the dd-cf-DNA levels did not exceed the threshold between POD 5 and 14. An aggregate of the data collected from the rejection group demonstrated elevated levels of dd-cf-DNA during the events of AR (red dots, Fig. 4c) relative to samples collected from the infection group (green dots, Fig. 4c) and the stable group (blue dots, Fig. 4c).

**Figure 5:**
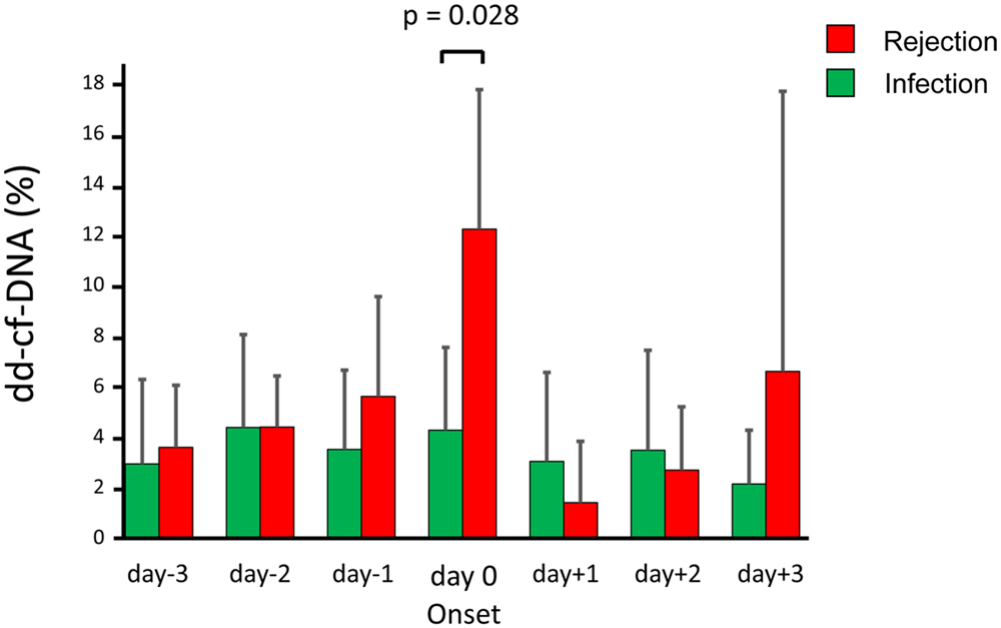
Plasma donor-derived cell-free DNA (dd-cf-DNA) levels during the same period around disease onset (day −3 to day +3 relative to the onset of acute rejection or infection) in the rejection group (Rejection, n = 4, red bars) and infection group (Infection, n = 5, green bars). The median levels were significantly higher in the rejection group as compared to those in the infection group (p = 0.028) on the day of the disease onset (day 0). There was no significant increase of the plasma dd-cf-DNA levels in the infection group.

To show a comparison of the plasma dd-cf-DNA levels between the rejection group and the infection group, the dd-cf-DNA levels between 3 days before and 3 days after the clinical onset of AR or infection (from day −3 to day +3, day 0 being the day of diagnosis of AR or infection) are shown in Fig. 5. The median levels in the rejection group increased gradually up to day 0 and were significantly higher than those in the infection group on day 0 (p = 0.028). The levels in the rejection group decreased after the administration of steroid pulse therapy for 3 consecutive days. Due to the persistence of high levels of dd-cf-DNA in the patients with AMR despite steroid pulse therapy (Patient 15), the dd-cf-DNA levels on day +3 were elevated in these patients (Fig. 4b).

## Discussion

In this study, the plasma dd-cf-DNA levels were significantly negatively correlated with the P/F ratio on POD 0 and POD 3 after LDLLT, suggesting that elevated dd-cf-DNA levels could be associated with decreased oxygenation of the transplanted lungs caused by primary graft dysfunction after LDLLT. Also, this study showed that the plasma dd-cf-DNA levels on the day of onset of AR were significantly higher than those in the stable and infection groups, suggesting that plasma dd-cf-DNA could be a potential biomarker of AR after LDLLT. To the best of our knowledge, this is the first report focusing on the postoperative dynamics of dd-cf-DNA after solid organ transplantation from living donors.

Recently, increased plasma levels of dd-cf-DNA in recipients were shown to be correlated with AR after solid organ transplantation from deceased donors, including heart^14^, lung^15^, liver^16^, and kidney^17^ transplantation. Consistent with these results, measurement of the plasma dd-cf-DNA could be useful for monitoring for lung tissue damage caused by primary graft dysfunction and early diagnosis of AR after LDLLT. However, the characteristics of LDLLT are such that there may be some difficulty in differentiating dd-cf-DNA by the droplet digital polymerase chain reaction (PCR) (ddPCR) assay in this study. In bilateral LDLLT, which involves two different living donors, identification of the SNPs from among three individuals, including one recipient and two living donors, was more challenging than that in the case of single LDLLT and cadaveric LT, which involves only a single donor. In single LDLLT, as is usual in cadaveric LT, it was simple to identify the target SNPs which were heterozygous in the donor and homozygous in both, but different, in the donor and the recipient. Theoretically, when the minor allele frequency (MAF) of SNPs is 0.5, the matching probability of this setting in single LDLLT and cadaveric LT would be 37.5%. By contrast, for the identification of the target SNPs of the first donor in bilateral LDLLT, the target SNPs should be heterozygous or homozygous in the first donor and different, but homozygous in both the second donor and the recipient. When the MAF is 0.5, the matching probability of this setting in bilateral LDLLT would be only 9.4%, much lower than that in the case of single LDLLT and cadaveric LT. In addition, DNA patterns of blood-relative donors in LDLLT are generally more similar to those of the recipients than is the case in cadaveric LT. Therefore, detection of dd-cf-DNA by ddPCR in LDLLT cases poses a bigger challenge than that in cases of cadaveric LT.

Although the dd-cf-DNA levels were found to be markedly increased immediately after cadaveric LT^15^, existence of a correlation between the plasma dd-cf-DNA levels and primary graft dysfunction has never been reported before. In this study, we showed that the increased levels of dd-cf-DNA were significantly correlated with decreased oxygenation on POD 0 and POD 3. Because the grade of primary graft dysfunction on POD 0 and POD 3 has been shown to have significant correlation with early outcomes after cadaveric LT^18^, we focused on the relationship between the dd-cf-DNA levels and primary graft dysfunction on POD 0 and POD 3 after LDLLT in this study. As compared to cadaveric LT, grafts from healthy donors for LDLLT are small, but ideal and similar in quality in LDLLT. Moreover, the ischemic time is shorter in LDLLT than that in cadaveric LT, because the operation in both the donor and recipient are simultaneously performed at the same hospital. Different from the use of extended-criteria donor lungs in cadaveric LT, these factors might provide favorable condition to evaluate postoperative oxygenation, presumably leading to the statistically significant difference in the degree of decreased oxygenation after LDLLT. In addition, the graft volume in LDLLT measured by pulmonary function testing and three-dimensional computed tomography, was not correlated with the plasma dd-cf-DNA levels in this study. However, the levels on POD 0 after LDLLT in this study were lower than that those after cadaveric LT described in the previous report (7.0% vs. 26%)^15^. The level of dd-cf-DNA immediately after LT could reflect the difference in the graft volume between the lobar lungs in LDLLT and the whole lungs in cadaveric LT, as well as the lower degree of graft injury in LDLLT, described above. These are consistent with the previous report that plasma dd-cf-DNA levels after cadaveric LT are correlated with the transplant tissue mass, and those after bilateral cadaveric LT are higher than those after single cadaveric LT^15^.

Consistent with a previous report regarding AR after cadaveric LT^15^, the plasma dd-cf-DNA levels were significantly higher on the day of onset of AR than those on the day of onset of infection or in the stable group after LDLLT. Interestingly, one patient with AMR showed higher levels of dd-cf-DNA than the other patients with AR. This is because AMR is known as steroid-refractory rejection and the lung tissue injury is more severe until additional treatment takes effect, including plasma exchange and rituximab therapy^19^. By contrast, in the infection group, the plasma dd-cf-DNA levels showed only mild elevation from the baseline, with no statistically significant difference as compared to those in the stable group in this study. The use of antibiotics in the early postoperative period after LDLLT might decrease the severity of infection and the plasma dd-cf-DNA levels, because severe pneumonia and sepsis have been shown to be associated with elevated dd-cf-DNA levels^20^. However, the significant difference of the plasma dd-cf-DNA levels between the AR and infection groups could contribute to early diagnosis and prompt treatment in the acute phase after LDLLT, so that subsequent complications can be minimized^21^. On the other hand, in the chronic phase after LT, differentiating AR from infection by monitoring of the plasma dd-cf-DNA levels appears to be difficult^15^. Therefore, further investigation is required to elucidate the role of plasma dd-cf-DNA measurement in the chronic phase after LDLLT.

Currently, LDLLT has been performed exclusively in Japan due to the severe donor shortage. To avoid unexpected bleeding and pneumothorax in the small grafts in LDLLT, AR after LDLLT has commonly been diagnosed clinically, without histological confirmation, for the past two decades in Japan. Our results indicate that measurement of the plasma dd-cf-DNA may be a desirable non-invasive technique for the diagnosis of AR after LDLLT, as well as for enhancement of the current diagnostic strategies for AR after LDLLT. Although transbronchial biopsy remains the gold standard for the diagnosis of AR after LT^22^, not only is it invasive, but it also has the drawbacks of sampling errors and variability in the pathologic interpretation^10,23–25^. Further detailed evaluation would be needed for plasma dd-cf-DNA measurement to become an alternative diagnostic approach to histological diagnosis in the future.

We applied the ddPCR technique to measure the plasma levels of dd-cf-DNA in this study. Currently, ddPCR^26^ and next-generation sequencing^27^ are available to quantify cf-DNA. Both techniques can provide precise quantification of dd-cf-DNA in the recipient; however, ddPCR is known as the more rapid and cost-effective method as compared to next-generation sequencing. Therefore, ddPCR is a suitable test method for early diagnosis in the clinical setting, that is, by monitoring the dd-cf-DNA levels for rejection and infection, in the early phase after LT. However, given the difficulty in detection of dd-cf-DNA after LDLLT as described above, next-generation sequencing might be more appropriate for identifying the target SNPs in three individuals, including one recipient and two living donors, involved in bilateral LDLLT.

Our study had several limitations. First, the number of cases of LDLLT enrolled in this study was small, because the number of donations from living donors is limited in Japan. Second, DNA analysis of brain-dead donors is not legally approved in Japan, and we could not compare the plasma dd-cf-DNA levels between patients who underwent LDLLT and those who underwent cadaveric LT. Third, because of the similar DNA patterns between the blood-relative donors and the recipients of LDLLT, detection of the target SNPs using a total of 35 SNPs resulted in the exclusion of 2 recipients of LDLLT from this study. Finally, because AR is diagnosed clinically without histological confirmation after LDLLT, and treated by steroid pulse therapy, other possible steroid-responsive pulmonary disorders could not be completely excluded. However, consistent with a previous report^14^, all patients clinically diagnosed as showing AR showed elevated plasma levels of dd-cf-DNA in this study, and monitoring of the plasma dd-cf-DNA levels could provide pertinent information for the diagnosis of AR even after LDLLT.

In conclusion, increased plasma levels of dd-cf-DNA were correlated with decreased oxygenation immediately and 72 hours after LDLLT, and the development of AR, but not infection, during the first 14 days after LDLLT. Measurement of the plasma dd-cf-DNA levels might be useful to monitor the severity of primary graft dysfunction after LDLLT, and plasma dd-cf-DNA could be used as a potential biomarker for the diagnosis of AR after LDLLT.

## Materials and Methods

### Patients

Between October 2011 and November 2016, 22 patients underwent LDLLT for various end-stage pulmonary diseases at Okayama University Hospital. In all patients, the lobar lungs were procured from living-donors at Okayama University Hospital. No organs were procured from prisoners in this study. Of these, 5 patients who did not provide consent for genotyping and 2 patients in whom SNPs could not be adequately identified due to the similar DNA typing patterns the between recipients and blood-relative donors, were excluded from this study. Of the remaining 15 patients, 8 patients underwent bilateral LDLLT from 16 living donors, and 7 patients underwent single LDLLT from 7 living donors. Nine of 15 patients were less than 18 years of age as shown in Table 1. The patients were divided according to the occurrence of clinical events between POD 0 and POD 14 after LDLLT: the 6 recipients who remained stable with no clinical signs of infection or AR were designated as the stable group, and 5 recipients who developed infection were designated as the infection group, and 4 recipients who showed clinical signs of AR and received steroid pulse therapy were designated as the rejection group (Fig. 1). The study protocol (No. 1601-030) was approved by the institutional review board of Okayama University Hospital. Written informed consent was obtained from the recipients ≥18 years of age, the parents of the pediatric recipients <18 years of age and the living donors. All methods were performed in accordance with the relevant guidelines and regulations.

### Operative procedures and perioperative care

LDLLT is considered for critically ill patients who meet the criteria for cadaveric LT, but cannot await cadaveric LT. Only blood relatives within the third degree or a spouse ≥20 years of age are accepted as living donors at our hospital. The size-matching protocol and procedures of LDLLT have been described previously^28^. The graft ischemic time was defined as the ischemic time of the second transplanted lung in bilateral LDLLT. The perioperative management has been described previously^7^. Briefly, all patients received triple immunosuppression therapy consisting of tacrolimus, mycophenolate mofetil and a steroid. Patients were assigned grades of primary graft dysfunction at four time-points according to the definition of primary graft dysfunction proposed by the International Society for Heart and Lung Transplantation: upon admission to the intensive care unit and at 24 hours, 48 hours and 72 hours after admission to the intensive care unit^29^. To avoid unexpected intragraft bleeding occurring as a result of the invasion associated with transbronchial biopsy, AR was routinely diagnosed on the basis of clinical and radiographic findings, including repeated bronchoscopies and computed tomography, without transbronchial biopsy, after LDLLT. Episodes of AR were treated by bolus intravenous methylprednisolone (5–10 mg/kg or 500 mg per day) for 3 consecutive days. AMR was diagnosed on the basis of allograft dysfunction, donor-specific anti–human leukocyte antigen antibodies, lung histology and C4d staining according to the consensus report of the International Society for Heart and Lung Transplantation^30^. Antimicrobial therapy was administered based on the antibiotic sensitivities of perioperative sputum cultures of the recipient.

### Sample collection and preparation

Pretransplant whole-blood samples were collected from the recipients and the living donors for SNP genotyping. Whole-blood samples were stored at −20 °C. DNA was extracted using a TaqMan Sample-to-SNP™ kit (Applied Biosystems, Foster City, CA, USA). For the first 14 days after LDLLT, 4 ml of peripheral blood samples from patients ≤12 years of age or 7 ml from patients >12 years of age were collected daily in EDTA tubes. The first blood sample on POD 0 was collected immediately after admission to the intensive care unit. Blood samples were centrifuged at 3,500 × g for 10 minutes. Plasma was transferred to microcentrifuge tubes and centrifuged at 16,000 × g for 10 minutes to remove residual cells. The two centrifugation steps were performed within 24 hours after the blood collection. Cell-free plasma was stored at −20 °C until further processing, and was frozen and thawed only once before the DNA extraction. The cf-DNA was extracted from 1 mL of plasma using the QIAamp Circulating Nucleic Acid Kit (QIAGEN, Hilden, Germany).

### SNP genotyping for donor-specific DNA

To identify the SNPs that differed between the recipient and living donor, a total of 35 different SNPs with a known and validated MAF between 0.4 and 0.5 in Japanese people were selected from the public database of the National Bioscience Database Center in Japan. SNP genotyping was performed in all 15 recipients and 23 living donors. Genomic DNA was isolated from the whole blood sample with TaqMan primers and probe (Applied Biosystems), and subsequently genotyping was performed by a TaqMan SNP genotyping assay using the StepOne™ real-time PCR system (Applied Biosystems) in a 96-well array plate that included four blank wells as negative controls. The PCR conditions consisted of an initial denaturation step at 95 °C for 10 minutes, 40 cycles at 92 °C for 15 seconds, and at 60 °C for 1 minute. The PCR products were analyzed using the StepOne™ Software Ver2.3 (Applied Biosystems). To assess the quality of the genotyping, the genotyping was conducted for all samples by using ddPCR and 100% agreement was obtained. For identification of the target SNPs between the recipient and the living donor, the living donors of the right graft were selected in the stable group, and the living donors who donated the graft that showed AR or infection were selected in the other two groups. In the single LDLLT cases, SNPs that were heterozygous in the donor, or homozygous in both but different between the donor and recipient were identified as donor-specific DNA. In the bilateral LDLLT cases, SNPs that were heterozygous or homozygous in the first donor and differently homozygous in both the second donor and the recipient were identified as donor-specific DNA.

### Quantification of the plasma dd-cf-DNA by ddPCR

After identification of the donor-specific genotype for each recipient, the dd-cf-DNA level was measured by ddPCR (QX200, Bio-Rad, Hercules, CA, USA), as described in a previous report^26^. The reactions were performed in 21-µl volumes of the reaction mixture consisting of up to 15 ng of extracted cf-DNA (8.8 µl), the ddPCR supermix for probe (10 µl), 0.5 µl of 30× TaqMan primers and probe set (TaqMan-validated SNP assays, Applied Biosystems, Foster City, CA, USA), and deionized distilled water (1.7 µl). The emulsified PCR reactions were run in a 96-well plate on a C1000 Touch thermal cycler. The plates were incubated at 95 °C for 10 minutes, followed by 40 cycles at 95 °C for 15 seconds, at 60 °C for 60 seconds, and a 10-minute incubation period at 98 °C. The plates were read on a Bio-Rad QX200 droplet reader using the QuantaSoft v1.4.0 software (Bio-Rad) to assess the number of droplets positive for the informative assays. For quantification of the minor allele fractional abundance, the embedded “Rare Event Detection” calculation was used, which takes the underlying Poisson distribution into account to calculate the template molecule concentration of either allele. These values are then used to express the minor allele abundance as a percentage of the total concentration. The final percentages of dd-cf-DNA (dd-cf-DNA/total cf-DNA) were obtained by summing the fractional abundances from the informative assays, where heterozygous SNP values were multiplied by two.

### Statistical analyses

All statistical analyses were performed using the JMP 11 software (SAS Institute Inc., Cary, NC, USA). Time-course of the dd-cf-DNA levels in the stable group are expressed as means ± standard deviations. Difference in the baseline characteristics among the groups were tested by the Kruskal-Wallis test for continuous variables, and Pearson’s chi-square test for categorical variables. Bivariate comparison of continuous variables between the two groups was performed using the Mann–Whitney U test. Associations between the plasma dd-cf-DNA levels and P/F ratio were tested by univariate regression analysis. Value of p < 0.05 were considered as denoting statistical significance.
